# Controlling the Go / No-Go decision threshold in the striatum

**DOI:** 10.1186/1471-2202-14-S1-P228

**Published:** 2013-07-08

**Authors:** Jyotika Bahuguna, Ad Aertsen, Arvind Kumar

**Affiliations:** 1 Bernstein Center Freiburg, University of Freiburg, 79104, Germany; 2 Neurobiology and Biophysics, Faculty of Biology, University of Freiburg, 79104, Germany; 3 Computational Biology, School of Computer Science and Communication, KTH-Stockholm, 10044, Sweden

## 

A typical Go/No-Go decision is thought to be implemented via the activation of direct and indirect pathways in the basal ganglia. Indeed, optogenetic activation of the direct pathway increased ambulation, whereas that of the indirect pathway induced freezing [1]. Striatal neurons participating in these two pathways express D1 and D2 type dopamine receptors [2]. Furthermore, D1 and D2 expressing MSNs also differ in their passive properties [2] and recurrent connectivity [3]. To understand striatal function it is, therefore, important to identify factors that regulate the balance of activity in D1 and D2 MSNs. Here we used both, a reduced firing rate model and numerical simulations of the striatal networks to study the dynamic balance of spiking activity in D1 and D2 MSNs.

Specifically, we show that: (i) Because D1 MSNs receive higher recurrent inhibition from FSIs [4] and D2 MSNs [3], they require a stronger cortical drive to overcome this inhibition. (ii) D1 and D2 firing rates change non-monotonically as a function of cortical input rates. For small cortical input rates, D1 MSNs have higher firing rates than D2 MSNs, due to the stronger synaptic input from cortex. For higher cortical input rates, D2 MSNs activity surpasses D1 MSN activity because cortical input rate is no longer sufficient to balance the strong inhibition coming from FSIs. The cortical rate at which D2 MSNs activity exceeds that of D1 MSNs is termed the decision threshold. (iii) The decision threshold depends on the strength of cortico-striatal synapses and the firing rate of FSIs. (iv) The STN could control the decision threshold via the massive pallidostriatal back-projections [5], via inhibition of the FSIs. (v) Finally, the difference between D1 and D2 firing rates is also modulated by the input correlations [6].

These observations help us to explain several experimental and behavioral findings involving the basal ganglia. The model suggest that under dopamine depletion conditions, even for weak cortical inputs, D2 MSNs activity is higher than D1 MSNs, which is consistent with the fact that Parkinson's disease (PD) patients have difficulty in initiating voluntary actions. We also observed that dopamine depletion reduced the parameter regime supporting D1 MSNs activation, by shifting the decision threshold towards lower cortical inputs. This suggests that under dopamine-depleted conditions, the striatum would require arbitration by the STN-GPe network, even for a low conflict task, providing a plausible explanation of increased reaction times in PD patients. Finally, increased activity in GPe under the influence of deep-brain stimulation (DBS) could also reduce the activity of FSIs on an average, thus shifting the decision threshold towards higher cortical input rates. Taken together, the model provides a mechanistic explanation of impulsive behavior in PD patients with DBS.
